# Impact of surgical versus spontaneous fetal reduction on maternal and neonatal outcomes after assisted reproductive technology: a single-center cohort study of 1, 451 singleton live births

**DOI:** 10.3389/fendo.2026.1923529

**Published:** 2026-10-26

**Authors:** Junzhai Song, Ting Zhang, Kaili Wang, Zhaozhao Liu, Canyu Li

**Affiliations:** Department of Obstetrics and Gynecology, The Third Affiliated Hospital of Zhengzhou University, Zhengzhou, Henan, China

**Keywords:** assisted reproductive technology, gestational diabetes mellitus, multifetal pregnancy reduction, preterm birth, spontaneous fetal reduction

## Abstract

**Objective:**

To compare maternal and neonatal outcomes among pregnancies with surgical reduction, spontaneous reduction, and initial singleton pregnancies following assisted reproductive technology (ART), and to identify independent risk factors for adverse obstetric outcomes.

**Methods:**

This retrospective cohort study included 1,451 singleton live births after ART at a single center from January 2018 to December 2023. Participants were divided into three groups: surgical reduction (n=128), spontaneous reduction (n=156), and initial singleton control group (n=1,167). Baseline characteristics, maternal complications, and neonatal outcomes were compared. Multivariate logistic regression was performed to identify independent risk factors.

**Results:**

Univariate analysis revealed significant intergroup differences in the incidence of gestational diabetes mellitus (GDM) and preterm birth. The GDM rate was highest in the spontaneous reduction group (26.92%), followed by the surgical reduction group (19.53%) and singleton controls (15.94%, *P* = 0.003). Preterm birth also differed significantly across groups (19.23% vs 14.06% vs 11.83%, *P* = 0.024). No group differences were detected for hypertensive disorders of pregnancy, placental complications, or congenital birth defects. After adjusting for maternal age, pre-pregnancy BMI, embryo type, No. of embryos transferred and embryo stage, multivariate logistic regression showed that surgical reduction (*OR* = 1.22, 95%CI 0.98-1.52, *P* = 0.069) and spontaneous reduction (*OR* = 1.39, 95%CI 0.98-1.98, *P* = 0.067) were not independent risk factors for GDM. Advanced maternal age (*OR* = 1.12, 95%CI 1.01-1.23, *P* = 0.028) and elevated pre-pregnancy BMI (*OR* = 1.11, 95%CI 1.01-1.21, *P* = 0.030) were the only independent predictors of GDM. For preterm birth, both surgical reduction (*OR* = 1.35, 95%CI 1.09-1.68, *P* = 0.007) and spontaneous reduction (*OR* = 1.72, 95%CI 1.20-2.45, *P* = 0.003) were independently associated with higher odds of preterm birth. Although the adjusted OR was numerically higher for spontaneous reduction than for surgical reduction, a direct comparison between the two methods showed no statistically significant difference.

**Conclusion:**

In ART singleton live births, a history of fetal reduction (surgical or spontaneous) was not independently associated with GDM, but both reduction modalities were independently associated with higher odds of preterm birth.

## Introduction

The widespread application of assisted reproductive technology (ART) has significantly improved the clinical pregnancy rate among infertile patients but has also led to a substantial increase in the incidence of multiple pregnancies (1). Multiple pregnancy is regarded as one of the major complications of ART, with maternal risks including gestational hypertension, gestational diabetes mellitus (GDM), placental disorders, and preterm delivery, threatening maternal and neonatal safety. Multifetal pregnancy reduction (MFPR) is widely adopted to reduce fetal number and mitigate perinatal risks for patients with high-order multiple gestations (2, 3).

MFPR is mainly categorized into two types: surgical reduction, which involves active transabdominal or transvaginal mechanical aspiration of embryonic tissue; and spontaneous reduction. Currently, whether there are differences in the effects of these two reduction modalities on subsequent pregnancy outcomes, and what the risk levels are compared with the initial singleton pregnancy population, remains lacking systematic investigation (4, 5).

It has been hypothesized that the two reduction modes may trigger divergent intrauterine inflammatory stimuli. Spontaneous reduction may generate sustained release of apoptotic embryonic and placental debris, potentially driving chronic low-grade systemic inflammation that could interfere with insulin sensitivity and weaken fetal membrane integrity. In contrast, surgical reduction removes excess embryos via controlled ultrasound-guided puncture and aspiration; intrauterine trauma and inflammatory activation may be transient and limited, without prolonged tissue breakdown. This proposed biological contrast provides the physiological rationale for the present comparative analysis of maternal glucose metabolism and gestational length. Notably, these inflammatory mechanisms were not directly measured in the present study and should be regarded as biological hypotheses rather than established explanations.

Previous studies have largely focused on the perinatal outcomes of surgical reduction, confirming that it can reduce the risks associated with multiple pregnancies (6, 7). However, most of these studies compared surgical reduction with unreduced multiple pregnancies rather than with the ideal singleton pregnancy controls. Moreover, existing studies often have insufficient control over confounding factors such as age, BMI, embryo type, and embryo stage, leading to ongoing controversy regarding the independent association between reduction modalities and adverse outcomes.

Given the above-mentioned biological speculations, the present study aimed to address the following research questions: Among ART-conceived pregnancies ending in singleton live birth, do pregnancies with spontaneous or surgical fetal reduction exhibit poorer obstetric outcomes compared with initial singleton pregnancies?

International reproductive societies including the American Society for Reproductive Medicine (ASRM) and European Society of Human Reproduction and Embryology (ESHRE) uniformly recommend elective single embryo transfer (eSET) as the first-line strategy to eliminate iatrogenic multiple pregnancies and their attendant complications. Limiting transferred embryo quantity is the fundamental preventive measure to avoid the need for MFPR or spontaneous reduction events, which aligns with the clinical implication of our study.

## Materials and methods

This study is a retrospective cohort study, with data sourced from the obstetrical clinical database of the Third Affiliated Hospital of Zhengzhou University. Data were collected from patients who received *in vitro* fertilization-embryo transfer or intracytoplasmic sperm injection at our hospital or other institutions between January 2018 and December 2023. Only those who achieved clinical pregnancy, and ultimately delivered singleton live births were included. This study adheres to the ethical principles of the Declaration of Helsinki regarding medical research involving human subjects. The study protocol was approved by the Ethics Committee of the Third Affiliated Hospital of Zhengzhou University. All raw clinical data were fully anonymized prior to statistical analysis to protect patient privacy.

This manuscript was drafted and reported in strict accordance with the Strengthening the Reporting of Observational Studies in Epidemiology (STROBE) statement for retrospective cohort studies.

Based on the number of gestational sacs observed by ultrasound in the first trimester and subsequent reduction status, the study subjects were divided into the following three groups (1): Surgical reduction group: 128 patients with initial twin pregnancies (two gestational sacs and two fetal heartbeats) who underwent active transvaginal surgical reduction to a singleton in the first trimester (2). Spontaneous reduction group: 156 women with initial twin pregnancies (2 gestational sacs and 2 fetal heartbeats) who experienced spontaneous cessation of development and resorption of one embryo without any medical intervention (3); Control group: 1,167 patients with initial singleton pregnancies (1 gestational sac and fetal heartbeat). Exclusion criteria included: complete miscarriage or cessation of all embryos in the first trimester, remaining twins or multiple pregnancies after reduction, presence of uterine malformations, chromosomal abnormalities, or severe maternal underlying diseases.

All surgical MFPR procedures were performed between gestational weeks 7-11 (early first trimester). The standardized transvaginal protocol was adopted: ultrasound-guided puncture of the gestational sac with full aspiration of embryonic tissue.

Two independent trained researchers extracted pre-ART infertility history, prenatal laboratory results, and delivery outcome data.

Definition of medical terms: Gestational hypertension is new-onset BP ≥140/90 mmHg after 20 weeks, resolving within 12 weeks postpartum; GDM is diagnosed at 24–28 weeks by 75g OGTT if fasting ≥5.1mmol/L,1h ≥10.0mmol/L, or 2h ≥8.5 mmol/L; placenta previa is placental coverage of the cervical os on ultrasound after 28 weeks; placenta accreta spectrum is abnormal trophoblastic invasion into the myometrium confirmed by imaging or pathology; placental abruption is premature separation of a normally implanted placenta after 20 weeks; and preterm birth is delivery <37 weeks. All diagnoses were based on internationally accepted clinical standards and confirmed by obstetrical specialists.

Statistical analyses were performed using SPSS 26.0 (IBM,USA) and R 4.2 software. For continuous variables following a normal distribution, data were expressed as mean ± standard deviation (Mean ± SD), and comparisons among the three groups were performed using one-way analysis of variance (ANOVA). For categorical variables, data were expressed as frequencies (percentages) [n (%)], and comparisons among the three groups were performed using the chi-square test (χ² test). Two binary multivariate logistic regression models were constructed for GDM and preterm birth respectively, with adjustment for baseline confounders including maternal age, pre-pregnancy BMI, No. of embryos transferred, embryo type (fresh/frozen-thawed), and embryo developmental stage (cleavage/blastocyst).

Covariate selection was guided by prior clinical knowledge and a predefined causal framework for ART-related adverse pregnancy outcomes, rather than merely based on statistical significance of between-group baseline comparisons. Although infertility duration showed statistically significant intergroup difference at baseline, it was not incorporated into the final multivariable models: sensitivity analyses demonstrated that adding infertility duration did not materially alter the main effect estimates for fetal-reduction-related variables. We acknowledge potential residual confounding caused by exclusion of this variable and other unavailable clinical covariates.

All logistic regression models were independently re-verified by two analysts; regression coefficients, standard errors, *OR*s, 95% CIs, and *P* values were cross-checked for internal consistency.

We acknowledge that gestational age at the time of spontaneous or surgical reduction was not collected in our dataset and could not be incorporated into the regression models; this represents a substantial limitation rather than a minor omitted covariate. Reduction timing may influence volume and duration of retained trophoblastic tissue, placental remodeling, and inflammatory exposure, and may modify subsequent obstetric outcomes. The absence of timing data directly limits the interpretation of hypothesized biological contrasts between spontaneous tissue resorption and surgical aspiration. This may constitute a residual confounder and is discussed as a limitation. Of note, only patients with twin pregnancies prior to reduction were enrolled in this study; therefore, the baseline gestational sac number was uniform (two sacs) across all participants, making adjustment for this variable unnecessary. All statistical tests adopted a two-sided α=0.05; *P* < 0.05 was defined as statistically significant.

## Results

As shown in **Table 1**, baseline characteristics significantly varied among the three groups in terms of age, duration of infertility, and number of embryos transferred, while no significant differences were observed in BMI, baseline FSH, type of infertility, embryo type, or embryo stage (*P* > 0.05).

**Table 1:** Basic parameters in surgical/spontaneous reduction patients and controls.

Parameters	Surgical reduction	Spontaneous reduction	Controls	*P*
N	128	156	1167	
Age (years)	31.28 ± 4.50	32.17 ± 4.41	31.20 ± 4.59	0.002
BMI (Kg/m^2^)	22.85 ± 3.62	22.61 ± 3.54	22.39 ± 3.75	0.067
Infertility duration (years)	5.40 ± 3.42	5.93 ± 3.88	5.32 ± 3.54	0.034
Basic FSH (mIU/mL)	6.20 ± 2.75	7.18 ± 3.60	6.31 ± 2.84	0.075
Infertility type				0.947
Primary	40.63% (52/128)	41.03% (64/156)	41.9% (489/1167)	
Secondary	59.38% (76/128)	58.97% (92/156)	58.1% (678/1167)	
Type of embryo				0.926
Fresh embryos	37.5% (48/128)	35.26% (55/156)	36.16% (422/1167)	
Frozen thawed embryos	62.5% (80/128)	64.74% (101/156)	63.84% (745/1167)	
Stage of embryo				0.951
Cleavage	54.69% (70/128)	54.49% (85/156)	55.61% (649/1167)	
Blastocyst	45.31% (58/128)	45.51% (71/156)	44.39% (518/1167)	

BMI, body mass index; FSH, follicular stimulation hormone.

Regarding maternal and neonatal outcomes, no significant differences were found among the three groups in the incidence of gestational hypertension, placenta previa, placenta accreta, or placental abruption. However, the incidence of GDM differed significantly between groups (P = 0.003): the spontaneous reduction group had the highest rate (26.92%), followed by the surgical reduction group (19.53%), and the initial singleton control group (15.94%). Preterm birth rates also showed a statistically significant difference (*P* = 0.024), with the spontaneous reduction group highest (19.23%), followed by surgical reduction (14.06%) and controls (11.83%). No significant difference was observed in the incidence of birth defects among the three groups (*P* = 0.635) (**Table 2**).

**Table 2:** Obstetrical and neonatal outcomes in surgical/spontaneous reduction patients and controls.

Parameters	Surgical reduction	Spontaneous reduction	Controls	*P*
N	128	156	1167	
Gestational hypertension	9.38% (12/128)	12.82% (20/156)	8.74% (102/1167)	0.255
Gestational diabetes	19.53% (25/128)	26.92% (42/156)	15.94% (186/1167)	0.003
Placenta previa	3.91% (5/128)	4.49% (7/156)	2.83% (33/1167)	0.458
Placenta accreta	10.16% (13/128)	12.18% (19/156)	10.28% (120/1167)	0.675
Placental abruption	2.34% (3/128)	3.21% (5/156)	2.14% (25/1167)	0.656
Preterm birth	14.06% (18/128)	19.23% (30/156)	11.83% (138/1167)	0.024
Birth defect	0.00% (0/128)	0.64% (1/156)	0.26% (3/1167)	0.635

In **Table 3**, multivariate logistic regression analysis identified that surgical reduction (*OR* = 1.22, 95% CI 0.98–1.52, *P* = 0.069) and spontaneous reduction (*OR* = 1.39, 95% CI 0.98–1.98, *P* = 0.067) were not independently associated with GDM, whereas age (*OR* = 1.12, 95% CI 1.01–1.23, *P* = 0.028) and BMI (*OR* = 1.11, 95% CI 1.01–1.21, *P* = 0.030) emerged as independent risk factors for GDM. For preterm birth, both surgical reduction (*OR* = 1.35, 95% CI 1.09–1.68, *P* = 0.007) and spontaneous reduction (*OR* = 1.72, 95% CI 1.20–2.45, *P* = 0.003) were independently associated with higher odds, although the adjusted OR for spontaneous reduction was numerically higher than that for surgical reduction, no formal direct comparison between the two methods was performed. Age was also independently associated with higher odds for preterm birth (*OR* = 1.25, 95% CI 1.09–1.42; *P* = 0.001), while BMI, No. of embryos transferred, embryo type, and embryo stage showed no significant independent associations.

**Table 3:** Association between multifetal pregnancy reduction methods and adverse pregnancy outcomes.

Parameters	Gestational diabetes					Preterm birth				
	β	SE	OR	95% CI	*P*	β	SE	OR	95% CI	*P*
Age	0.11	0.050	1.12	1.01-1.23	0.028	0.22	0.067	1.25	1.09-1.42	0.001
BMI	0.10	0.046	1.11	1.01-1.21	0.030	0.05	0.029	1.05	0.99-1.11	0.085
No. of embryos transferred	0.06	0.038	1.06	0.99-1.14	0.118	0.14	0.081	1.15	0.98-1.35	0.084
Type of embryo	-0.22	0.160	0.80	0.59-1.10	0.169	-0.13	0.097	0.88	0.73-1.06	0.180
Stage of embryo	0.16	0.140	1.17	0.89-1.54	0.253	-0.05	0.049	0.95	0.86-1.05	0.311
MFPR methods (Model I)										
No (controls)			1.00	Reference				1.00	Reference	
Surgical	0.20	0.110	1.22	0.98-1.52	0.069	0.30	0.111	1.35	1.09-1.68	0.007
Spontaneous	0.33	0.180	1.39	0.98-1.98	0.067	0.54	0.182	1.72	1.20-2.45	0.003
MFPR methods (Model II)										
No (controls)	-0.20	0.110	0.82	0.66-1.02	0.069	-0.30	0.111	0.74	0.60-0.92	0.007
Surgical			1.00	Reference				1.00	Reference	
Spontaneous	0.13	0.211	1.14	0.75-1.72	0.535	0.24	0.213	1.27	0.84-1.93	0.259

MFPR, Multifetal pregnancy reduction; BMI, body mass index; OR, odds ratio; CI, confidence interval; SE, standard errors; Model I and Model II are identical in the statistical model, adjustment variables, and calculation method; the only difference lies in the reference group.

In addition, a direct comparison using surgical reduction as the reference (Model II) showed no statistically significant difference between spontaneous and surgical reduction for preterm birth (OR 1.27, 95% CI 0.84–1.93, *P* = 0.259), GDM (OR 1.14, 95% CI 0.75–1.72, *P* = 0.535). Thus, although both reduction methods increased the risk of preterm birth relative to the singleton control group, their effects did not differ significantly from each other.

## Discussion

In recent years, the number of infertile couples has been increasing steadily. To improve clinical pregnancy rates in ART, controlled ovarian hyperstimulation and multiple-embryo transfer are commonly employed; however, this practice has led to a progressive rise in associated complications, the most common of which is multiple gestation. Multiple pregnancies significantly increase the risk of maternal and perinatal complications, including gestational hypertension, miscarriage, preterm birth, and fetal growth restriction (FGR), thereby elevating overall pregnancy risks and posing serious threats to maternal and neonatal health (8).

MFPR serves as a remedial strategy to reduce fetal numbers in multiple gestations and improve pregnancy outcomes; nevertheless, the retained fetuses may still be at risk of miscarriage and preterm birth (9). Luo et al. through a large retrospective analysis, reported a 17.1% incidence of spontaneous reduction in first-trimester twin pregnancies, with over 80% occurring before 12 weeks of gestation, and suggested that this phenomenon has limited adverse effects on pregnancy outcomes and does not increase the risk of adverse pregnancy outcomes (10). However, conflicting evidence has indicated that the surviving fetus following reduction may still experience unfavorable pregnancy outcomes. Moreover, studies have shown that ART patients with multiple pregnancies who undergo early single-fetal reduction continue to exhibit higher rates of gestational complications and preterm delivery compared to those with initial singleton pregnancies (11).

Our study stratified reduction subtypes and incorporated a large primary singleton control group to disentangle independent risks of surgical versus spontaneous reduction, adjusting for major maternal and embryo confounders to reduce baseline imbalance bias. Fresh/frozen embryo ratio and cleavage/blastocyst distribution were balanced across groups, minimizing confounding from embryo quality and allowing focused analysis of reduction-specific intrauterine inflammatory effects.

Univariate analysis demonstrated substantially higher crude GDM rates in both reduction groups compared with primary singletons, with spontaneous reduction showing the highest prevalence. Previous literature confirms multiple gestation aggravates maternal insulin resistance and elevates GDM susceptibility (12). Pathophysiologically, it has been proposed that sustained resorption of apoptotic embryonic tissue in spontaneous reduction may release pro-inflammatory cytokines and induce chronic low-grade inflammation and peripheral insulin resistance; surgical reduction may also trigger transient inflammatory stress from puncture trauma and tissue aspiration.

Crucially, after multivariate adjustment for age and BMI, neither surgical nor spontaneous reduction was independently associated with GDM. This suggests that the elevated crude GDM prevalence observed in reduction groups may be largely attenuated by baseline maternal metabolic characteristics rather than reduction-induced inflammation, although residual confounding cannot be excluded.

In our multivariable analysis, maternal age and elevated pre-pregnancy BMI were the factors most strongly associated with gestational glucose dysregulation. The observed attenuation of association after covariate adjustment is observational and cannot prove that inflammatory burden from fetal reduction only adds marginal metabolic stress that lacks statistical independence after accounting for pre-existing insulin resistance; inflammatory mediators and insulin resistance were not directly measured in this cohort. Any interpretation linking inflammation to GDM risk remains speculative. These findings do not support inflammatory debris as an independent causal mediator of GDM risk in our cohort, though the observational design and limited sample size preclude definitive causal inference.

No significant intergroup differences were observed in placenta-related complications or gestational hypertension. We detected no statistically significant group-level associations for these outcomes in the present cohort. This observation cannot establish that these complications are primarily determined by maternal vascular conditions and implantation sites rather than reduction-related factors; mechanistic explanations remain hypothetical. Nonetheless, some studies have reported that the incidence of hypertensive disorders of pregnancy is substantially higher in reduced pregnancies compared to initial singletons, with even greater risk when reduction is performed before 24 weeks of gestation (13).

Univariate testing confirmed significantly elevated preterm birth rates in reduction cohorts, with spontaneous reduction demonstrating the highest crude risk. After full confounder adjustment, both reduction modalities were independently associated with higher odds of preterm delivery. In addition, a direct comparison between the two reduction methods showed no significant difference for preterm birth or GDM). Thus, although both methods increased the risk of preterm birth relative to the singleton control group, their effects did not differ significantly from each other.

Surgical MFPR is well-established to reduce preterm risk relative to unreduced multiple gestations, yet few studies quantified residual preterm risk versus primary singleton pregnancies, and the potentially greater adverse impact of spontaneous reduction is frequently underrecognized. It has been proposed that spontaneous reduction, which typically occurs at gestational weeks 6–9, may release inflammatory debris and cytokines into the uterine cavity, potentially disrupting fetal membrane integrity and stimulating uterine smooth muscle contraction, thereby increasing premature rupture of membranes and spontaneous preterm labor risk (14). However, these inflammatory mechanisms were not directly measured in the present study. In contrast, ultrasound-guided surgical reduction enables precise, controlled elimination of excess embryos via aspiration; inflammatory activation may be briefer and more localized, potentially leading to milder residual preterm risk relative to spontaneous reduction. However, the invasive puncture procedure still generates minor endometrial injury, explaining the modestly elevated preterm odds compared with unmanipulated primary singleton pregnancies. No statistically significant difference in congenital malformation rates was observed across groups, with zero structural anomalies in the surgical reduction cohort. However, given the very small number of congenital anomalies overall, these findings cannot establish absence of teratogenic risk; they only indicate that no statistically significant association was detected in our cohort, consistent with Guo et al. (15).

The ultimate clinical goal of ART is delivery of a healthy term neonate. Interpretations should be restricted to pregnancies ending in singleton live birth after ART; we cannot draw conclusions for pregnancies ending in miscarriage, fetal loss or persistent multiple gestations. Consistent with ASRM and ESHRE international guidelines (16, 17), strict limitation of ovulation stimulation and universal promotion of elective single embryo transfer (eSET) remain the primary preventive strategy to avoid multiple gestations and subsequent fetal reduction events. Given the high proportion of advanced-age infertile patients under China’s three-child policy, standardized pre-transfer counseling regarding multiple gestation risks and eSET recommendations is clinically essential.

Our findings are broadly consistent with those of Wang et al., who also reported an increased risk of preterm birth after fetal reduction (18). However, unlike their study, we did not find that reduction was an independent risk factor for GDM after multivariable adjustment; advanced maternal age and elevated pre-pregnancy BMI were the main predictors. This discrepancy may be due to differences in sample size, inclusion criteria (we included only singleton live births), baseline characteristics, and adjustment strategies. Wang et al. also reported reduced live birth rate and birth weight after reduction and recommended SET, whereas our conclusions are more conservative. These differences may also reflect variations in reduction timing, indications, and study design.

This study has several notable limitations that must be acknowledged to avoid overinterpretation of results. First, single-center retrospective design with limited subgroup sample sizes restricts external generalizability; multi-center prospective cohorts are required to validate our findings. All study subjects were twin-origin before reduction; outcomes cannot be generalized to higher-order multifetal pregnancy reduction cases. The relatively wide 95% confidence intervals observed in the multivariate regression models reflect statistical uncertainty driven by small sample sizes within the two reduction subgroups, which represents a major statistical limitation of the present analysis.

Of particular importance, we lacked data on gestational age at reduction. Surgical reduction was performed 7-11 weeks, while exact timing of spontaneous reduction was unavailable. Timing may affect retained trophoblast volume, duration of tissue resorption and inflammatory exposure, which are core components of our proposed biological hypothesis. Furthermore, because only pregnancies resulting in singleton live births were included, exclusion of miscarriages, fetal losses, and persistent multiple pregnancies may introduce selection bias; therefore, our conclusions should be restricted to singleton live births following ART. Moreover, we lack long-term developmental follow-up data for surviving infants, limiting conclusions regarding childhood neurodevelopmental outcomes after fetal reduction.

In addition, inflammatory mediators and insulin-resistance markers were not measured in this study. Proposed inflammatory-driven biological pathways are hypothetical interpretations rather than conclusions directly supported by our clinical association data.

In conclusion, fetal reduction was associated with higher unadjusted rates of GDM and preterm birth. After adjustment, neither reduction method independently predicted GDM, whereas advanced age and elevated BMI did; however, both remained independently associated with preterm birth. Single embryo transfer therefore remains the optimal strategy to prevent multiple gestations and their complications.

## Data Availability

The raw data supporting the conclusions of this article will be made available by the authors, without undue reservation.
